# Supplementary material for: Regulation of Carotenoid Biosynthesis and Degradation in Lettuce (Lactuca sativa L.) from Seedlings to Harvest
Source: Int J Mol Sci. 2023 Jun 18;24(12):10310. doi: 10.3390/ijms241210310 (PMC10298985; doi:10.3390/ijms241210310)
Supplement: Supplementary file 1 [file ijms-24-10310-s001.zip › ijms-2399257-supplementary.pdf]

Supplementary material

Regulation of carotenoid biosynthesis and degradation in lettuce (*Lactuca sativa* L.) from seedlings to harvest

Galina Brychkova[1*], Cleiton Lourenço de Oliveira[1,2] , Luiz Antonio Augusto Gomes[3], Matheus de Souza Gomes[4], Antoine Fort[1,5], Alberto Abrantes Esteves-Ferreira[6], Ronan Sulpice[6], Peter McKeown[1], Charles Spillane[1*]

Supplementary Tables

Supplementary Table S1. Primers sequences (designed by Quantprime) used for qRT-PCR analysis.

Locus	Foward primer	Reverse primer	Accession number
UBQ	5'-CCATTTGGTCCTCCGTCTCC-3'	5'-CGAGGGTTCTGCCGTCTTCT-3'	DW141651
DXS	5'-CGCCATTGATGACAGACCCAG-3'	5'-GCCCTTCCAGCATTATTCGC-3'	AB205044
DXR	5'-AGAAACGAATCTTTGGTTGAAG-3'	5'-TCACACAATCAGGATGACGG-3'	AB205045
IPI	5'-TCACTTGACGGAGAAGATTGAG-3'	5'-CCATACCAAAGGAAATGTCACC-3'	AB205048
GGPS	5'-AATTCGGTAAATCAAGCCCTAG-3'	5'-ATACACAGCATCGGTCGGAC-3'	AB205047
GGPPS	5'-AACGCCACCCACAATCCATGAAG-3'	5'-ATGCAGAGAATGGGCCTCACAC-3'	DY975567
PSY1	5'-ACGACATCGTACACCATCTGCTC-3'	5'-TTCCAGGGTTGTGGTGGCTAAC-3'	DY974614
PSY2	5'-GCTTGACGCTGCTTTATCAGAC-3'	5'-TCTTCAGATCCATCCTCATTCC-3'	AB205050
PDS	5'-TAGCTGATGCAGGTCACAAGCC-3'	5'-TTCCAAGCTGCCACCTTTCCAC-3'	DY972145
ZDS	5'-ATCCACCTCATGCCCTTGATCC-3'	5'-TATCATAGGTGCTGGCCTTGCTG-3'	DY960874
CRTISO	5'-ATCTGTGATGTTGCGATTCAGC-3'	5'-ACGGTGGTTGGATCGGGTATC-3'	AB205043
LCY-β1	5'-AGGAAGATCAGGGCCAATGAGTCC-3'	5'-TCTTGCTGCAGAGTCAGCCAAAC-3'	DY968060
LCY-β2	5'-AAGGGTCTCGTGGTGGATC-3'	5'-TGAGGTGAAGGGTCAATCG-3'	AB205041
LCY-ε	5'-TCACACATCGTGCTAATGGATC-3'	5'-CCGTTGAAGATAAAGAAGACCC-3'	AB205046
CHY	5'-TAACCGGAAGGAGCGTGAACTG-3'	5'-TTTGCCTGCACTCCTCTTGGAC-3'	DY970341
CHYβ	5'-TCCGAACGCTTTACTTACCTTG-3'	5'-GAACATCTCCACCCTCCATTTG-3'	AB205042
VDE	5'-ACTCGCAACAATCGTCCTGAC-3'	5'-GGGCACACATTTCTTTCGG-3'	AB205051
ZEP	5'-GTTGGGTATCGGGTGTTCTTGG-3'	5'-GGCTGGTTCGTTGTGAAATGC-3'	AB205053

Supplementary Table S2. Regression analysis of factors affecting β -carotene accumulation. **A.** without considering plant genotype, plant developmental stages or leaves position.

	β	SE	STD β	t	p	95.0% CI	df	F	p
(Constant)	-70.29	19.29		-3.64	0.00	-108.66, -31.93	4, 85	21.01	<0.001
Lutein	5.03	0.67	0.59	7.46	0.00	3.69, 6.37			
Chla	0.08	0.05	0.55	1.50	0.14	-0.03, 0.18			
Chlb	0.09	0.05	0.42	1.67	0.09	-0.02, 0.20			
ChlT	-0.05	0.05	-0.56	-1.03	0.31	-0.15, 0.05			

Model's AIC: 927.87; BIC: 942.87

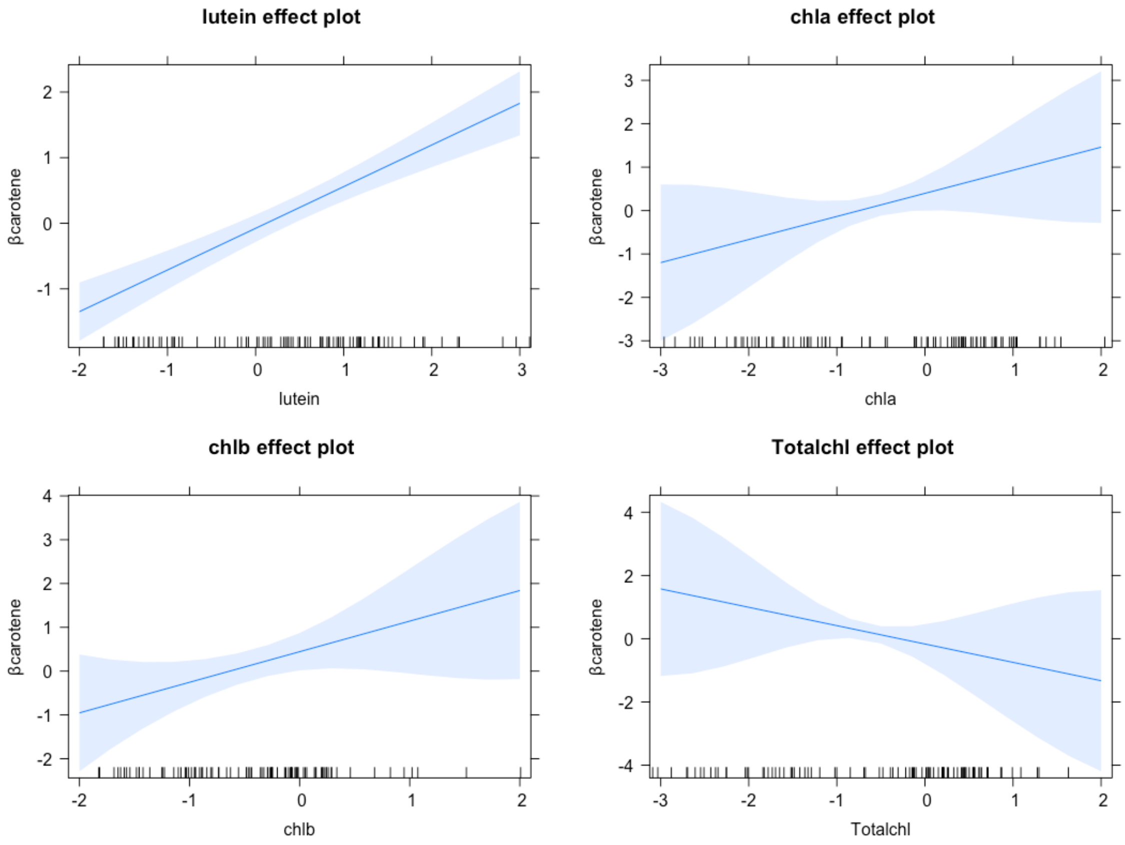

Supplementary Table S2. Regression analysis of factors affecting β-carotene accumulation **B.** accounting role of genotype, sampling time and leave position.

	β	SE	STD β	t	p	95.0% CI	df	F	p
(Constant)	-68.29	18.85		-3.62	0.00	-105.79, -30.78	7, 89	39.65	<0.001
Variety	-4.53	1.93	-0.14	-2.34	0.02	-8.37, -0.68			
Sampling time (Ts)	-66.39	7.72	-0.89	-8.60	0.00	-81.74, -51.04			
Leaves position (Lp)	57.91	8.44	0.51	6.86	0.00	41.11, 74.70			
Lutein	10.42	0.84	1.22	12.40	0.00	8.74, 12.09			
Chla	0.04	0.04	0.29	1.12	0.27	-0.03, 0.11			
Chlb	0.10	0.04	0.49	2.74	0.01	0.03, 0.18			
ChlT	-0.07	0.04	-0.78	-2.07	0.04	-0.15, -0.01			

Model's AIC: 644.06; BIC: 719.06

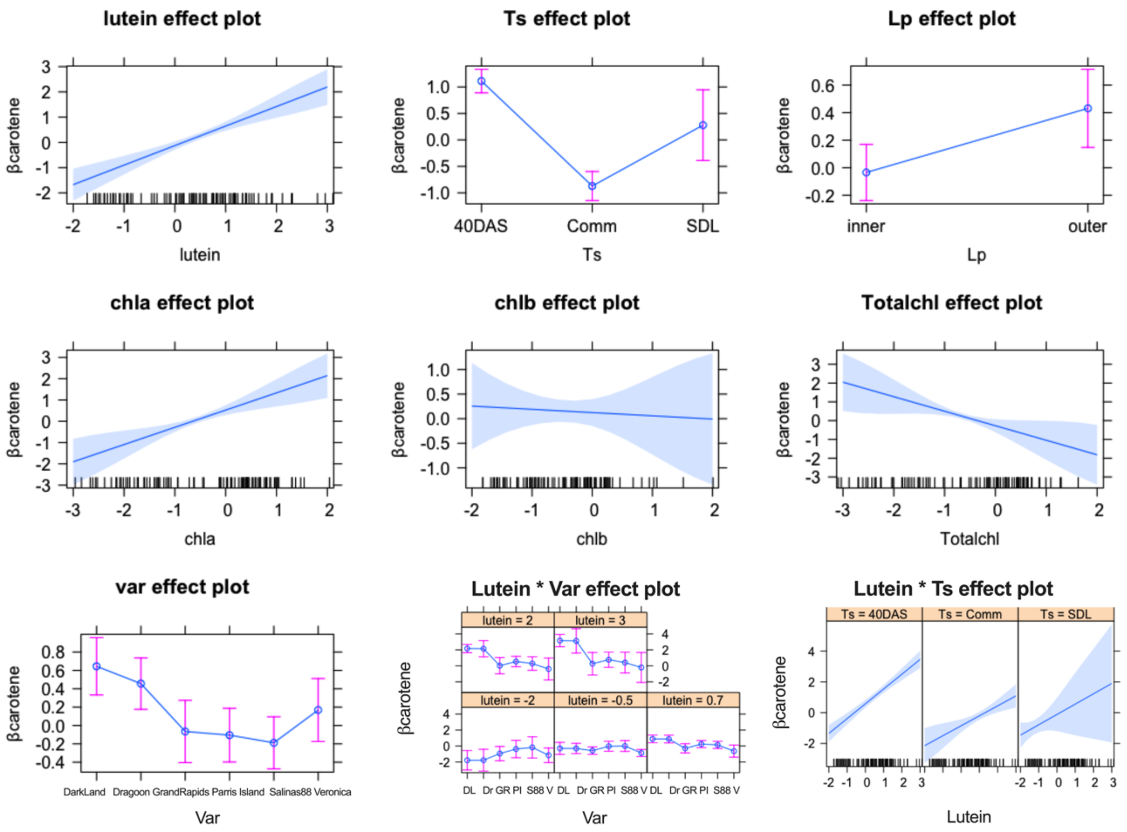

Supplementary Table S3. Pearson's correlation (r) comparing relative gene expression with total chlorophyll, lutein and β-carotene in inner and outer lettuce leaves at seedling stage, 40 days after sowing (DAS) stage and commercial size stage.

		Seedling			40 DAS inner			40 DAS outer			Commercial inner			Commercial outer		
**Gene**	**Comm[1]**	**Chl T**	**Lut[2]**	**β-car[3]**	**Chl T**	**Lut**	**β-car**	**Chl T**	**Lut**	**β-car**	**Chl T**	**Lut**	**β-car**	**Chl T**	**Lut**	**β-car**
DXS	Inner	0.56	0.64	0.41	0.11	0.69	0.5	-0.61	0.01	-0.2	-0.85	-0.31	-0.4	-0.90**	-0.44	-0.51
	Outer	0.21	0.43	0.07	-0.1	0.66	0.29	-0.23	0.12	0.09	-0.35	-0.21	-0.08	-0.55	-0.37	-0.29
DXR	Inner	-0.17	-0.4	-0.55	-0.39	-0.68	-0.83	-0.67	-0.39	-0.66	-0.26	-0.16	-0.4	0.1	-0.19	0.13
	Outer	0.23	-0.02	0.15	-0.06	-0.4	-0.22	-0.3	-0.1	-0.1	0.6	0.11	0.45	-0.09	-0.14	0
IPI	Inner	0.63	0.02	0.17	-0.04	-0.64	-0.5	0.92**	0.65	0.55	-0.23	0.06	-0.12	-0.43	-0.68	-0.47
	Outer	-0.11	-0.2	-0.4	-0.04	-0.54	-0.33	0.54	0.61	0.36	0.51	0.49	0.73	0.12	-0.46	0.05
GGPS	Inner	-0.33	-0.36	-0.33	-0.48	-0.87	-0.73	-0.48	-0.91	-0.78	-0.67	-0.25	-0.25	-0.54	-0.35	-0.44
	Outer	-0.9	-0.36	-0.67	-0.67	-0.9	-0.83	-0.14	-0.77	-0.4	0.09	-0.19	0.17	-0.27	0.03	0.09
GGPPS	Inner	0.53	0.43	0.25	0.37	0.73	0.69	-0.46	-0.18	-0.46	-0.34	-0.4	-0.35	-0.15	-0.11	-0.45
	Outer	0.92**	0.59	0.72	0.71	0.7	0.79*	0.25	0.22	0.4	0.57	-0.24	0.34	-0.29	0	-0.29
PSY1	Inner	0.71	0.72	0.65	0.82*	0.85*	0.89**	0.07	-0.08	-0.2	0.37	0.43	0.18	0.66	0.7	0.85
	Outer	0.92**	0.78*	0.84*	0.74	0.77	0.73	0.61	0.3	0.57	0.85*	0.54	0.65	0.54	0.72	0.71
PSY2	Inner	0.5	0.34	0.34	0.66	0.16	0.37	-0.66	-0.66	-0.76	-0.28	0.27	0.29	-0.04	-0.4	-0.4
	Outer	-0.42	0.23	-0.27	0.15	0.1	0.03	0.23	-0.43	0.04	0.33	0.49	0.73	0.52	-0.1	0.3
PDS	Inner	-0.41	-0.72	-0.77	-0.11	-0.54	-0.19	-0.13	-0.47	-0.5	-0.11	-0.55	-0.49	0.5	0.02	-0.1
	Outer	0.15	-0.4	-0.04	-0.03	-0.59	-0.24	0.22	-0.12	0.12	0.45	-0.34	0.12	0.49	-0.05	-0.03
ZDS	Inner	-0.1	-0.46	-0.47	-0.52	-0.32	-0.4	0	-0.35	-0.43	0.05	-0.3	-0.14	0.64	0.41	0.4
	Outer	0.38	-0.13	0.22	-0.43	-0.52	-0.54	0.28	-0.01	0.16	0.59	-0.3	0.19	0.29	0.56	0.38
CRTISO	Inner	0.47	0.39	0.46	-0.32	-0.41	-0.56	-0.63	-0.81	-0.88	0.7	0.82*	0.79*	-0.93**	-0.92**	-0.86*

	Outer	-0.13	0.45	0.14	-0.75	-0.38	-0.7	-0.15	-0.48	-0.21	0.81*	0.76*	0.76*	-0.35	-0.76*	-0.37
LCY-β1	Inner	0.42	0.39	0.26	0.32	0.26	0.13	-0.16	0.04	-0.24	0.36	0.35	0.03	0.01	0.21	0.08
	Outer	0.98***	0.53	0.77*	0.96***	0.46	0.76*	0.39	0.45	0.54	0.53	0.36	0.27	0.56	0.62	0.80*
LCY-β2	Inner	0.73	0.7	0.65	-0.42	-0.44	-0.72	-0.58	-0.8	-0.93	0.77*	0.85*	0.91**	0.03	-0.23	-0.11
	Outer	-0.16	0.52	-0.03	-0.25	-0.41	-0.46	-0.39	-0.76	-0.66	0.55	0.63	0.5	0.24	0.08	0.31
LCY-ε	Inner	0.72	0.54	0.39	0.4	0.01	0.19	-0.67	-0.45	-0.69	-0.42	-0.12	-0.42	-0.31	-0.73	-0.69
	Outer	-0.05	0.42	-0.09	-0.08	0.08	-0.02	-0.4	-0.16	-0.2	0.37	0.2	0.41	-0.39	-0.56	-0.49
CHY	Inner	-0.1	-0.32	-0.37	0.23	-0.12	0.21	-0.15	-0.37	-0.62	-0.31	-0.21	-0.51	0.29	0.08	0.12
	Outer	0.66	-0.2	0.26	-0.09	-0.45	-0.36	0.01	0.45	0.3	0.18	-0.14	-0.05	-0.36	0.15	-0.17
CHY-β	Inner	0.68	0.23	0.08	0.31	0.01	-0.16	-0.75	-0.71	-0.78	-0.22	0.07	-0.28	-0.36	-0.47	-0.28
	Outer	0.22	0.16	-0.1	0.75	0.18	0.39	-0.27	-0.72	-0.47	0.41	0.29	0.39	-0.12	-0.2	0.08
VDE	Inner	0.51	0.11	0.17	0.27	-0.27	-0.09	-0.79	-0.57	-0.81	0.08	0.49	0.56	-0.2	-0.41	-0.55
	Outer	-0.2	0.11	-0.19	0.05	-0.15	-0.07	-0.22	-0.36	-0.21	0.59	0.63	0.86*	0.35	-0.03	0.25
ZEP	Inner	0.56	0.42	0.58	0.54	0.12	0.19	0.09	0.28	0	-0.02	0.11	-0.16	-0.16	-0.48	-0.26
	Outer	0.42	0.55	0.57	0.7	0.27	0.49	0.66	0.63	0.74	0.67	0.25	0.46	-0.08	-0.26	-0.03

[1] Commercial size; [2] Lutein; [3] β-carotene; * Significant ($P < 0.05$); ** Significant ($P < 0.01$); *** Significant ($P < 0.001$).

Supplementary Table S4. An expression correlation analysis using *LsLCY-β1* & *LCY-β2* as the driver genes for β–carotene biosynthesis with all other carotenoid biosynthesis pathway genes.

Control Variables	Gene	DXS	DXR	IPI	GGPS	GGPPS	PSY1	PSY2	PDS	ZDS	CRTISO	LCY-ε	CHYε	CHYβ	VDE	ZEP
LCY-β1 & LCY-β2	DXS	1	-0.296	0.276	0.269	0.147	0.053	0.331	0.134	0.166	0.217	0.520**	-0.097	0.865**	0.367	0.221
	DXR	-0.296	1	-0.15	0.023	-0.08	-0.448*	0.141	-0.237	-0.322	0.103	-0.079	0.075	-0.259	0.433*	0.37
	IPI	0.276	-0.15	1	0.307	0.188	0.419*	0.706**	0.581**	0.447*	0.740**	0.113	0.032	0.136	0.397*	0.495**
	GGPS	0.269	0.023	0.307	1	0.248	0.102	0.708**	0.336	0.104	0.318	0.570**	0.011	0.508**	0.334	0.166
	GGPPS	0.147	-0.08	0.188	0.248	1	0.422*	0.077	0.367	0.269	0.164	0.169	0.325	0.081	-0.085	0.071
	PSY1	0.053	-0.448*	0.419*	0.102	0.422*	1	0.141	0.774**	0.773**	0.167	0.048	0.587**	0.051	-0.261	-0.129
	PSY2	0.331	0.141	0.706**	0.708**	0.077	0.141	1	0.451*	0.181	0.666**	0.381*	-0.104	0.460*	0.563**	0.385*
	PDS	0.134	-0.237	0.581**	0.336	0.367	0.774**	0.451*	1	0.794**	0.445*	0.284	0.549**	0.174	0.092	0.141
	ZDS	0.166	-0.322	0.447*	0.104	0.269	0.773**	0.181	0.794**	1	0.189	0.062	0.601**	0.146	-0.039	0.025
	CRTISO	0.217	0.103	0.740**	0.318	0.164	0.167	0.666**	0.445*	0.189	1	0.382*	-0.153	0.103	0.607**	0.794**
	LCY-ε	0.520**	-0.079	0.113	0.570**	0.169	0.048	0.381*	0.284	0.062	0.382*	1	0.02	0.532**	0.306	0.312
	CHYε	-0.097	0.075	0.032	0.011	0.325	0.587**	-0.104	0.549**	0.601**	-0.153	0.02	1	-0.081	-0.12	-0.043
	CHYβ	0.865**	-0.259	0.136	0.508**	0.081	0.051	0.460*	0.174	0.146	0.103	0.532**	-0.081	1	0.316	-0.032
	VDE	0.367	0.433*	0.397*	0.334	-0.085	-0.261	0.563**	0.092	-0.039	0.607**	0.306	-0.12	0.316	1	0.776**
	ZEP	0.221	0.37	0.495**	0.166	0.071	-0.129	0.385*	0.141	0.025	0.794**	0.312	-0.043	-0.032	0.776**	1

Note. Pink colour means Pearson correlation is >0.75. **. Correlation is significant at 0.01 level. *. Correlation is significant at 0.05 level.

Supplementary Table S5. An expression correlation analysis using *CHYε* as the driver gene for lutein biosynthesis to determine the level of co-expression that CHYε shares with all of the other carotenoid biosynthesis pathway genes.

Control Variables	Genes	DXS	DXR	IPI	GGPS	GGPPS	PSY1	PSY2	PDS	ZDS	CRTISO	LCY-β1	LCY-β2	LCY-ε	CHYβ	VDE	ZEP
CHYε	DXS	1	0.169	0.502**	0.440*	-0.026	-0.164	0.583**	0.356	0.238	0.487**	0.776**	0.614**	0.868**	0.970**	0.578**	0.567**
	DXR	0.169	1	-0.014	0.089	-0.169	-0.643**	0.195	-0.221	-0.399*	0.143	0.342	0.14	0.221	0.171	0.371*	0.327
	IPI	0.502**	-0.014	1	0.545**	0.188	0.303	0.811**	0.756**	0.587**	0.816**	0.2	0.584**	0.544**	0.470*	0.660**	0.710**
	GGPS	0.440*	0.089	0.545**	1	0.269	0.031	0.818**	0.565**	0.288	0.638**	0.106	0.596**	0.648**	0.556**	0.634**	0.559**
	GGPPS	-0.026	-0.169	0.188	0.269	1	0.33	0.148	0.26	0.151	0.234	-0.23	0.1	0.035	-0.041	0.049	0.126
	PSY1	-0.164	-0.643**	0.303	0.031	0.33	1	0.047	0.518**	0.580**	0.105	-0.256	-0.116	-0.179	-0.162	-0.233	-0.172
	PSY2	0.583**	0.195	0.811**	0.818**	0.148	0.047	1	0.703**	0.429*	0.883**	0.192	0.813**	0.757**	0.652**	0.837**	0.788**
	PDS	0.356	-0.221	0.756**	0.565**	0.26	0.518**	0.703**	1	0.731**	0.715**	0.072	0.481**	0.487**	0.394*	0.480**	0.489**
	ZDS	0.238	-0.399*	0.587**	0.288	0.151	0.580**	0.429*	0.731**	1	0.464*	-0.042	0.321	0.237	0.243	0.275	0.292
	CRTISO	0.487**	0.143	0.816**	0.638**	0.234	0.105	0.883**	0.715**	0.464*	1	0.092	0.819**	0.716**	0.494**	0.850**	0.924**
	LCY-β1	0.776**	0.342	0.2	0.106	-0.23	-0.256	0.192	0.072	-0.042	0.092	1	0.238	0.604**	0.738**	0.183	0.212
	LCY-β2	0.614**	0.14	0.584**	0.596**	0.1	-0.116	0.813**	0.481**	0.321	0.819**	0.238	1	0.829**	0.651**	0.779**	0.808**
	LCY-ε	0.868**	0.221	0.544**	0.648**	0.035	-0.179	0.757**	0.487**	0.237	0.716**	0.604**	0.829**	1	0.883**	0.717**	0.747**
	CHYβ	0.970**	0.171	0.470*	0.556**	-0.041	-0.162	0.652**	0.394*	0.243	0.494**	0.738**	0.651**	0.883**	1	0.596**	0.528**
	VDE	0.578**	0.371*	0.660**	0.634**	0.049	-0.233	0.837**	0.480**	0.275	0.850**	0.183	0.779**	0.717**	0.596**	1	0.901**
	ZEP	0.567**	0.327	0.710**	0.559**	0.126	-0.172	0.788**	0.489**	0.292	0.924**	0.212	0.808**	0.747**	0.528**	0.901**	1

Note: Pink colour means Pearson correlation is >0.75. **. Correlation is significant at 0.01 level.
*. Correlation is significant at 0.05 level.

Supplementary Table S6. Lettuce plants used in the experiments.

Genotype	Type	Genotype origin	Seeds origin
DarkLand	Romaine	USA	UFLA Germplasm Bank
Dragoon	Mini Romaine	USA	UFLA Germplasm Bank
Grand Rapids	Loose leaf	USA	UFLA Germplasm Bank
Parris Island	Romaine	USA	UFLA Germplasm Bank
Salinas 88	Crisp Head	USA	UFLA Germplasm Bank
Verônica	Loose Leaf	Brazil	UFLA Germplasm Bank

Supplementary Figures

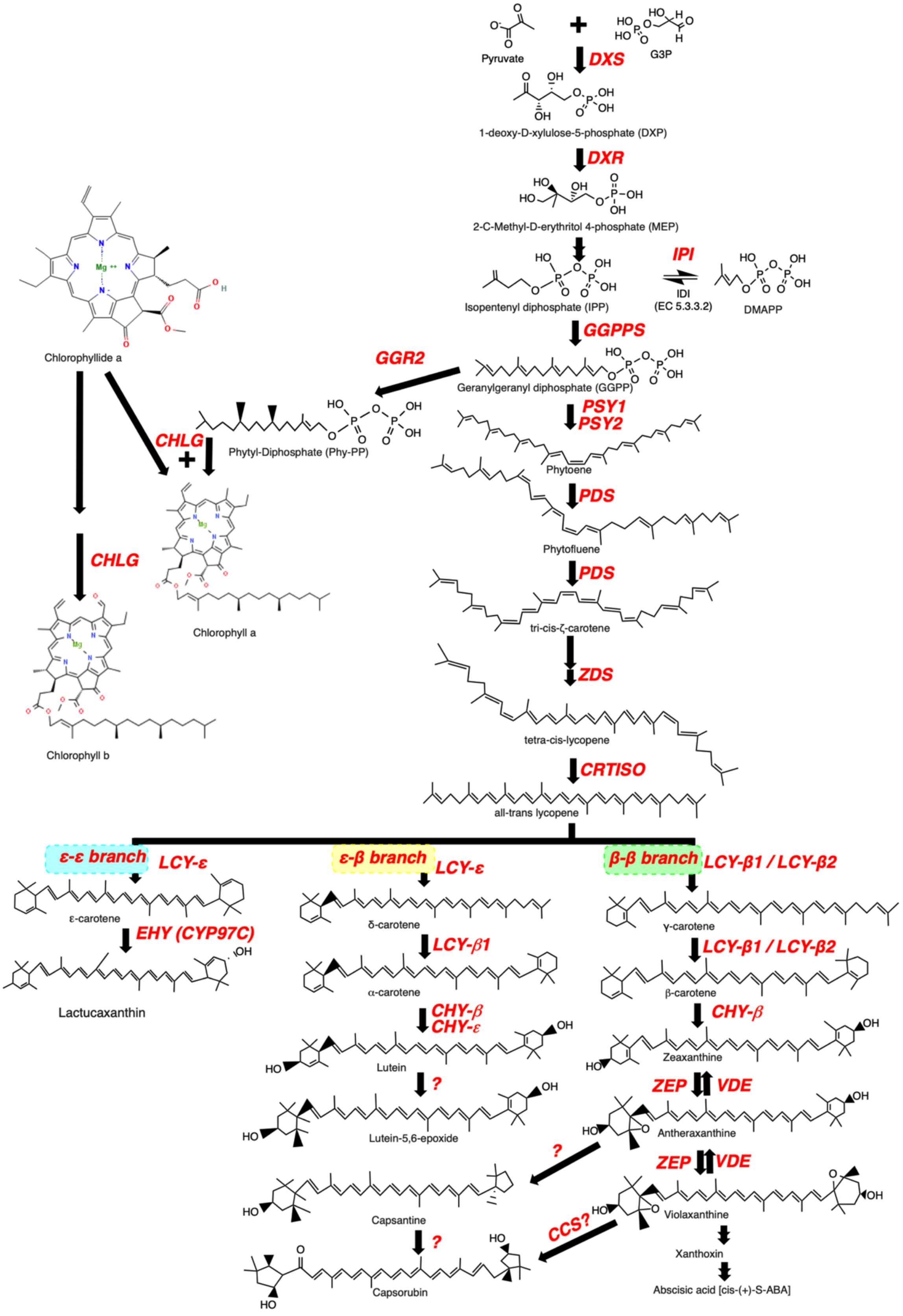
Pyruvate
G3P
DXS
1-deoxy-D-xylulose-5-phosphate (DXP)
DXR
2-C-Methyl-D-erythritol 4-phosphate (MEP)
IPI
Isopentenyl diphosphate (IPP)
IDI
(EC 5.3.3.2)
DMAPP
GGPPS
Geranylgeranyl diphosphate (GGPP)
GGR2
Phytyl-Diphosphate (Phy-PP)
Chlorophyllide a
CHLG
Chlorophyll a
CHLG
Chlorophyll b
PSY1
PSY2
Phytoene
PDS
Phytofluene
PDS
tri-cis-ζ-carotene
ZDS
tetra-cis-lycopene
CRTISO
all-trans lycopene
ε-ε branch
LCY-ε
ε-carotene
EHY (CYP97C)
Lactucaxanthin
ε-β branch
LCY-ε
δ-carotene
LCY-β1
α-carotene
CHY-β
CHY-ε
Lutein
?
Lutein-5,6-epoxide
β-β branch
LCY-β1 / LCY-β2
γ-carotene
LCY-β1 / LCY-β2
β-carotene
CHY-β
Zeaxanthine
ZEP
VDE
Antheraxanthine
ZEP
VDE
Violaxanthine
Xanthoxin
Abscisic acid [cis-(+)-S-ABA]
?
Capsantine
?
CCS?
Capsorubin

Supplementary Figure S1. Schematic representation of the non-mevalonate (methylerythritol 4-phosphate, MEP) chloroplasts pathway and mevalonate (cytosolic) pathway of carotenoid biosynthesis in *Lactuca sativa*, *Asteraceae*. The enzymes' names are highlighted in bold. Abbreviations: GA3P, glyceraldehyde-3-phosphate; DXS, 1-deoxyxylulose 5-phosphate synthase; DOXP, D-1-deoxyxylulose-5-phosphate; DXR, 1-deoxyxylulose 5-phosphate reductoisomerase; MEP, 2-C-methyl-D-erythritol-2,4- cyclodiphosphate; IPP, isopentenyl diphosphate; IPI, IPP isomerase; GGPP, geranylgeranyl diphosphate; GGPS, GGPP synthase; PSY1, phytoene synthase; PDS, phytoene desaturase; ZDS, ζ-carotene desaturase; CRTISO, carotenoid isomerase; LCY-B, lycopene β-cyclase; LCY-E, lycopene ε-cyclase; CHYB, β-ring hydroxylase; CHYE, ε-ring hydroxylase; ZEP, zeaxanthin epoxidase; VDE, violaxanthin deepoxidase; EHY, ε-carotene hydroxylase; CHLG, Chlorophyll synthase; GGR2, geranylgeranyl reductase2.

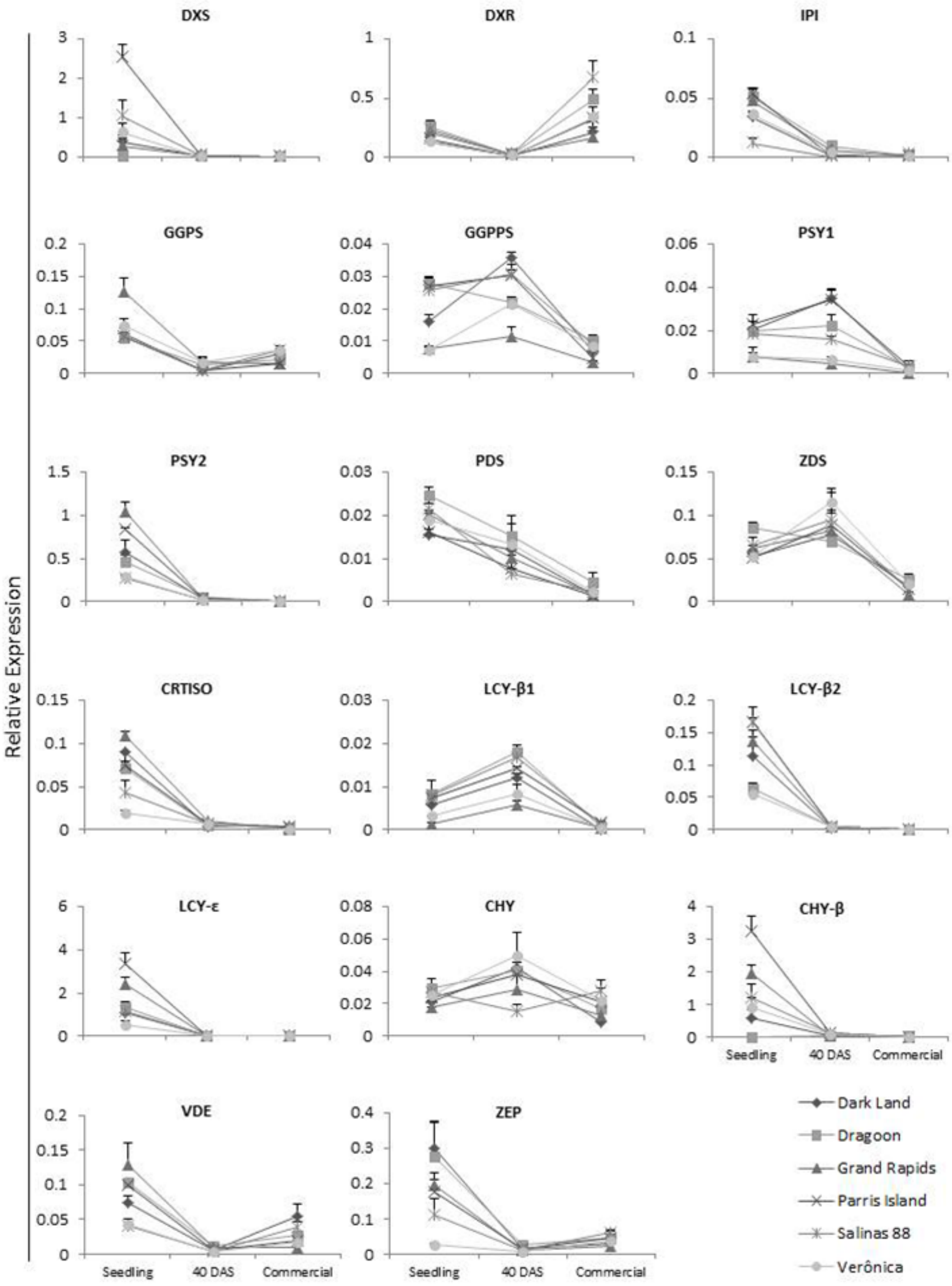

Supplementary Figure S2. Relative expression of lettuce carotenoid biosynthetic genes in inner leaves of different lettuce cultivar measured at seedling stage, 40 days after sowing (DAS), and commercial size (60 DAS) stages. Gene expression was measured relative to *UBIQUITIN*. Error bars are standard deviation of the mean from three biological replicates.

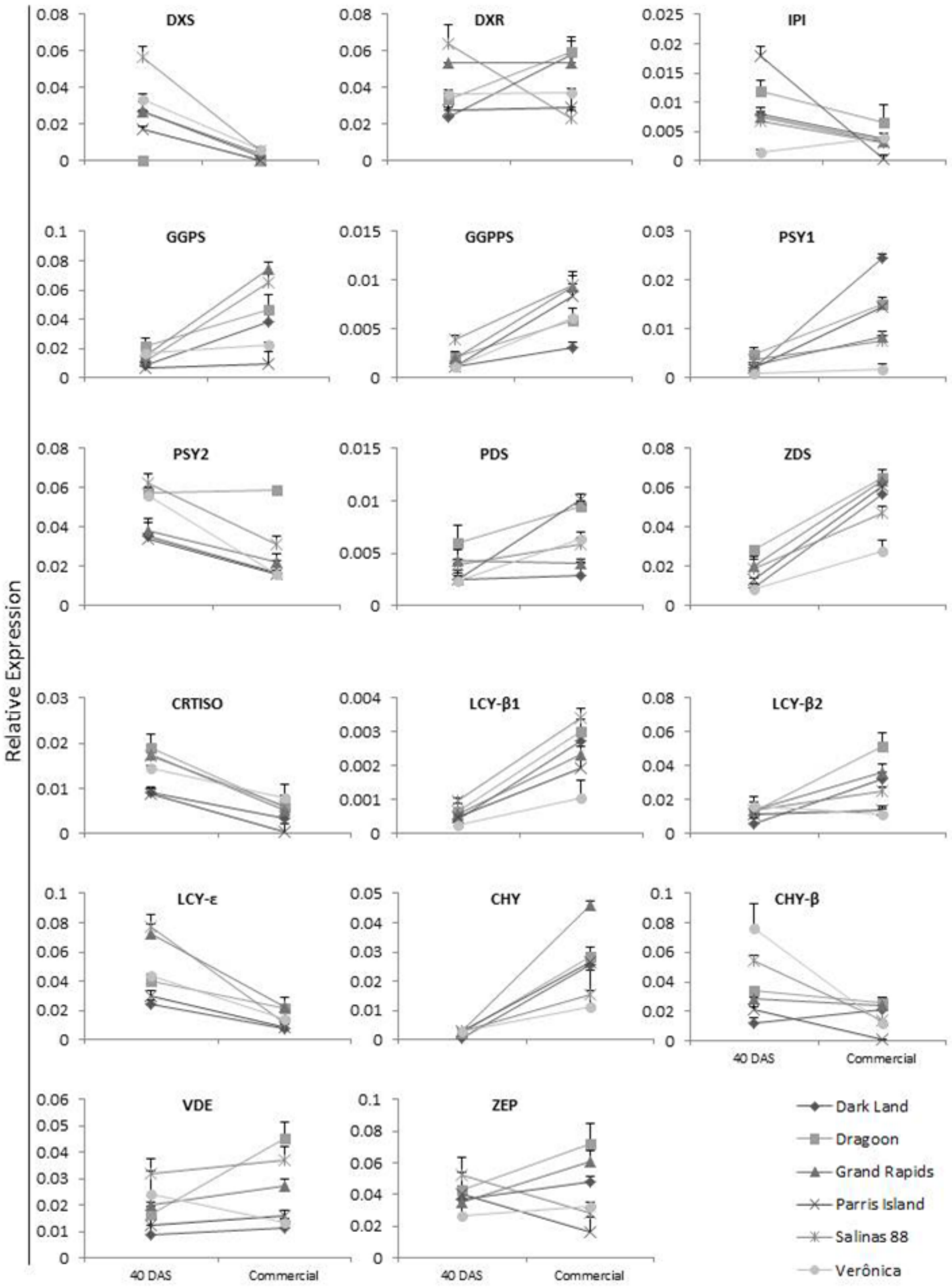

Supplementary Figure S3. Relative expression of lettuce carotenoid biosynthetic genes in outer leaves of different lettuce cultivar measured at 40 days after sowing (DAS) and commercial size (60 DAS) stages. Gene expression was measured relative to *UBIQUITIN*. Error bars are standard deviation of the mean from three biological replicates.

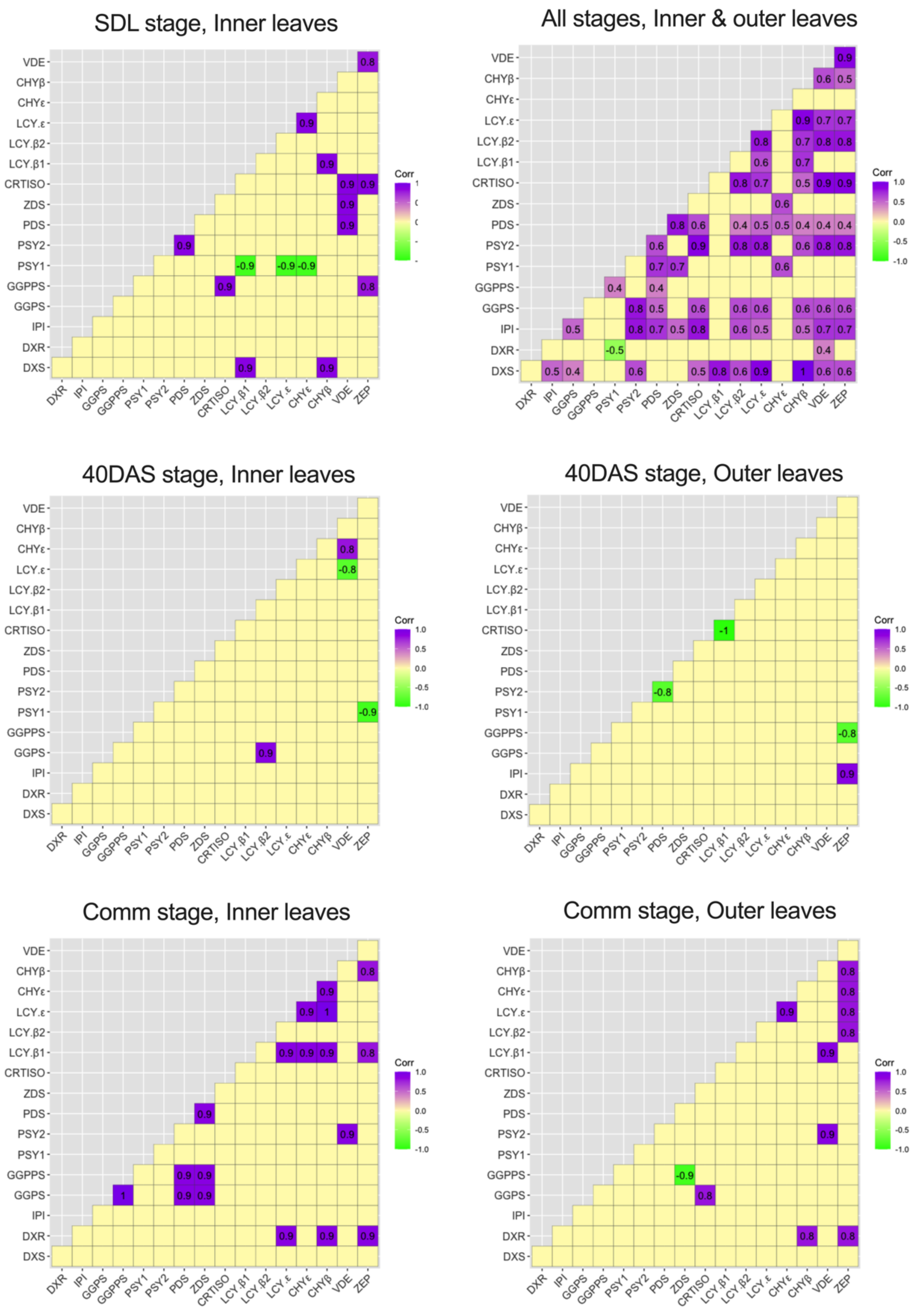

Supplementary Figure S4. Pearson correlation analysis of Relative expression of lettuce carotenoid biosynthetic genes in outer leaves of different lettuce cultivar measured 20days after sowing (SDL), at 40days after sowing (DAS) and commercial size (60DAS) stages. Only significant correlation is presented with Pearson correlation results. Correlation is significant at 0.05 level.

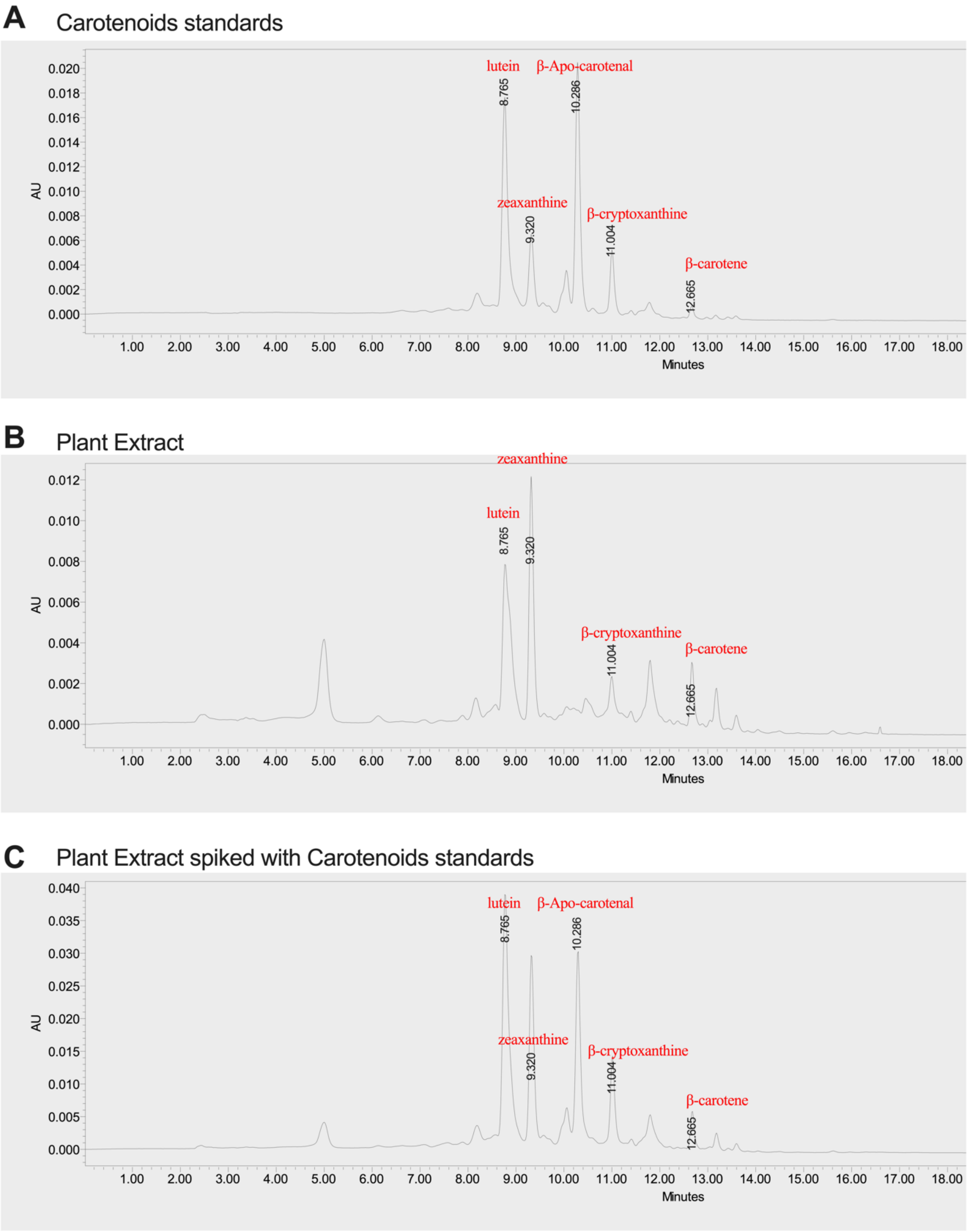

Supplementary Figure S5. Carotenoids HLPC profile. A. Standards used to analyse carotenoids content in leaves. B. Plant extracts carotenoi profile. C. Plant extract spiked with carotenoids standards.
